# DRP-1 functions independently of mitochondrial structural perturbations to facilitate BH3 mimetic-mediated apoptosis

**DOI:** 10.1038/s41420-019-0199-x

**Published:** 2019-07-17

**Authors:** Mateus Milani, Alison J. Beckett, Aoula Al-Zebeeby, Xu Luo, Ian A. Prior, Gerald M. Cohen, Shankar Varadarajan

**Affiliations:** 1 Department of Molecular and Clinical Cancer Medicine, Institute of Translational Medicine, University of Liverpool, Liverpool, Ashton Street, Liverpool, L69 3GE UK; 2 Department of Cellular and Molecular Physiology, Institute of Translational Medicine, University of Liverpool, Liverpool, Ashton Street, Liverpool, L69 3GE UK; 3 Eppley Institute for Research in Cancer and Allied Diseases, Fred and Pamela Buffett Cancer Center, University of Nebraska Medical Center, Omaha, NE 68198 USA; 4 Department of Molecular and Clinical Pharmacology, Institute of Translational Medicine, University of Liverpool, Liverpool, Ashton Street, Liverpool, L69 3GE UK

**Keywords:** Cell death, Organelles

## Abstract

Maintenance of mitochondrial integrity is critical for normal cellular homoeostasis. Most cells respond to stress stimuli and undergo apoptosis by perturbing mitochondrial structure and function to release proteins, such as cytochrome *c*, which are essential for the execution of the intrinsic apoptotic cascade. Cancer cells evade these events by overexpressing the anti-apoptotic BCL-2 family of proteins on mitochondrial membranes. Inhibitors of the anti-apoptotic BCL-2 family proteins, also known as BH3 mimetics, antagonise the pro-survival functions of these proteins and result in rapid apoptosis. Although the precise mechanism by which BH3 mimetics induce apoptosis has been well characterised, not much is known in terms of the structural changes that occur in mitochondria during apoptosis. Using a panel of highly selective BH3 mimetics and a wide range of cell lines, we demonstrate that BH3 mimetics induce extensive mitochondrial fission, accompanied by swelling of the mitochondrial matrix and rupture of the outer mitochondrial membrane. These changes occur in a BAX/ BAK-dependent manner. Although a major mitochondrial fission GTPase, DRP-1, has been implicated in mitochondrial apoptosis, our data demonstrate that DRP-1 might function independently/downstream of BH3 mimetic-mediated mitochondrial fission to facilitate the release of cytochrome *c* and apoptosis. Moreover, downregulation of DRP-1 prevented cytochrome *c* release and apoptosis even when OPA1, a protein mediating mitochondrial fusion, was silenced. Although BH3 mimetic-mediated displacement of BAK and other BH3-only proteins from BCL-X_L_ and MCL-1 was unaffected by DRP-1 downregulation, it prevented BAK activation significantly, thus placing DRP-1 as one of the most critical players, along with BAX and BAK, that governs BH3 mimetic-mediated cytochrome *c* release and apoptosis.

## Introduction

Most chemotherapeutic agents kill cancer cells by executing the intrinsic apoptotic pathway, which is characterised by mitochondrial outer membrane permeabilization (MOMP), release of cytochrome *c* from the inner mitochondrial membrane (IMM) and formation of the apoptosome that activates the initiator and effector caspases. MOMP is regulated by the BCL-2 family, whereby BAX and BAK, undergo specific conformational changes to form oligomeric pores that insert into the outer mitochondrial membrane (OMM) to release cytochrome *c*^1,2^. Activation of BAX and BAK is achieved by several pro-apoptotic BH3-only members, which are generally rendered ineffective by sequestration with specific anti-apoptotic BCL-2 family of proteins, such as BCL-2, BCL-X_L_ and MCL-1^3,4^. These anti-apoptotic proteins are highly expressed in many cancers and inhibitors known as BH3 mimetics have been designed to target them in order to displace the BH3-only proteins, activate BAX and BAK, thereby inducing MOMP and apoptosis of cancer cells^5^.

ABT-737, and its orally available analogue, ABT-263 (Navitoclax) were the first bona fide BH3 mimetics developed to target BCL-2, BCL-X_L_ and BCL-w^6,7^. Subsequently, BH3 mimetics that specifically target BCL-2 (ABT-199 (Venetoclax), S55746), BCL-X_L_ (A-1331852) and MCL-1 (A-1210477, S63845, AMG 176 and AZD5991) have been synthesised^8–14^. These inhibitors, as single agents, have demonstrated much promise in treating a wide variety of haematological malignancies, and have had limited success in combination with conventional chemotherapy against several solid tumours^8,12–17^. BH3 mimetics induce apoptosis primarily by targeting protein–protein interactions between the anti- and pro-apoptotic BCL-2 family members^18^. Subsequently, BH3 mimetics have been shown to induce significant structural changes in the mitochondria, ranging from mitochondrial matrix swelling to discontinuities in the OMM, upstream of caspase activation^19,20^. Furthermore, BAX and BAK localise to the breakpoints in OMM and may facilitate cytochrome *c* release at such breakpoints^19^. Although BCL-2 family members have been implicated in regulating mitochondrial membrane dynamics and functions^21–24^, putative inhibitors of MCL-1 have often resulted in extensive mitochondrial fission in various cell lines^25–27^. The regulation of this fission and its relationship to BH3 mimetic-mediated apoptosis remains to be determined.

Mitochondrial structure is maintained through an intricate balance between the activities of several fusion and fission proteins, which belong to a conserved family of GTPases that reside in the OMM or IMM. Mitofusins 1 and 2 (MFN1/ 2) and optic atrophy 1 (OPA1) are essential for mitochondrial fusion, whereas dynamin related protein 1 (DRP-1) is essential for mitochondrial fission^28^. Defects in mitochondrial fusion and fission have been implicated in a range of pathophysiological conditions including poor brain development, optic atrophy, cardiomyopathy and neurodegenerative diseases^29,30^. Mounting evidence now suggests the involvement of several members of BCL-2 family members, particularly MCL-1, in the regulation of mitochondrial structure and function^22–25,29^. However, the mechanism by which MCL-1 regulates mitochondrial membrane dynamics and the potential cross-talk with its conventional role in antagonising apoptosis remain to be characterised.

In this study, we use a panel of highly selective BH3 mimetics together with cell lines that depend on specific BCL-2 family members for survival to demonstrate that BH3 mimetics induce significant ultrastructural mitochondrial changes upstream of caspase activation. DRP-1 plays a role downstream of these changes but upstream of MOMP to facilitate cytochrome *c* release and apoptosis, following exposure to BH3 mimetics.

## Results

### BH3 mimetics induce marked mitochondrial structural changes

**Fig. 1:**
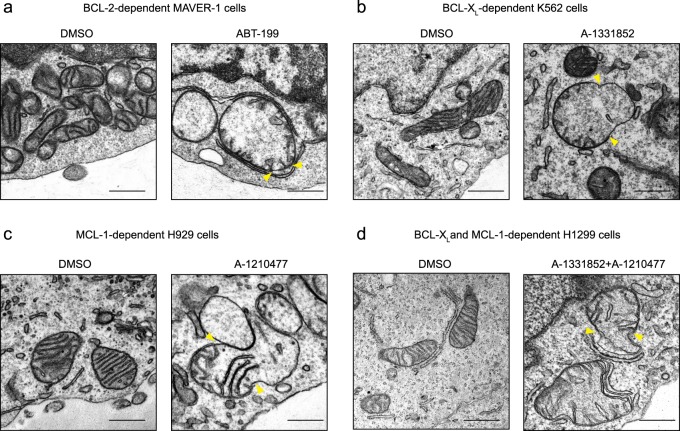
BH3 mimetics induce marked ultrastructural changes in mitochondria of different cells. **a** MAVER-1, **b** K562, **c** H929 and **d** H1299 cells were exposed to Z-VAD.fmk (30 µM) for 0.5 h, followed by ABT-199 (100 nM), A-1331852 (100 nM), A-1210477 (10 µM), or a combination of A-1331852 (100 nM) and A-1210477 (10 µM), respectively, for 4 h and assessed for mitochondrial structural changes by electron microscopy. Yellow arrowheads indicate regions of breaks at the outer mitochondrial membrane. Scale bars: 500 nm

Previously, we have reported that BH3 mimetics induce a novel paradigm of apoptosis characterised by marked ultrastructural changes in the mitochondria, involving the loss of mitochondrial cristae and the appearance of breaks in the OMM, resulting from mitochondrial matrix swelling^19,20,31^. In cell lines that depend for survival almost exclusively on BCL-2 (MAVER-1), BCL-X_L_-(K562) and MCL-1 (H929)^32^, exposure to the relevant BH3 mimetics, such as ABT-199, A-1331852 and A-1210477, respectively, resulted in similar mitochondrial matrix swelling and rupture of the OMM (Fig. 1a–c). Such mitochondrial changes were also evident in H1299 cells following exposure to a combination of A-1331852 and A-1210477, as these cells depend on both BCL-X_L_ and MCL-1 for survival (Fig. 1d). These mitochondrial ultrastructural changes were independent of effector caspases, as they were observed in cells pre-treated with Z-VAD.fmk, a broad-spectrum caspase inhibitor (Fig. 1). Exposure of the different cells to their appropriate BH3 mimetic resulted in mitochondrial membrane depolarisation, loss of cytochrome *c* and induction of apoptosis, as assessed by phosphatidylserine externalisation (Supplementary Fig. S1). Exposure of the cells to Z-VAD.fmk almost completely inhibited BH3 mimetic-mediated apoptosis, assessed by PS externalisation, whereas little if any inhibition of cytochrome *c* release was observed (Supplementary Fig. S1). Taken together these results suggested that the mitochondrial structural changes occurred upstream of effector caspase activation and accompanied cytochrome *c* release, as well as a loss of mitochondrial membrane potential.

### BH3 mimetic-mediated mitochondrial perturbations occur in a BAX/BAK-dependent manner

**Fig. 2:**
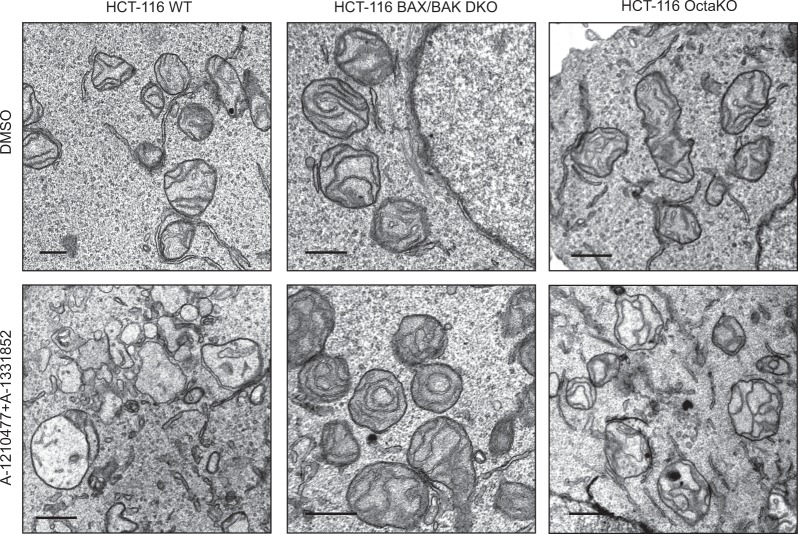
BH3 mimetics disrupt mitochondria in a BAX- and BAK-dependent but BH3-independent manner. HCT-116 WT, DKO (BAX/BAK deficient) and OctaKO cells were exposed to Z-VAD.fmk (30 µM) for 0.5 h, followed by a combination of A-1331852 (100 nM) and A-1210477 (10 µM) for 4 h and assessed for mitochondrial structural changes by electron microscopy. Scale bars: 500 nm

To assess whether BAX and BAK play crucial roles in BH3 mimetic-mediated ultrastructural changes in mitochondria, we exposed HCT-116 WT and BAX/BAK double knock-out (DKO) cells to a combination of A-1331852 and A-1210477, as HCT-116 cells also depend on both BCL-X_L_ and MCL-1 for survival^33^. Exposure of the HCT-116 WT cells to the BH3 mimetics resulted in significant mitochondrial matrix swelling accompanied by a loss of mitochondrial cristae, although rupture of the OMM was not readily apparent (Fig. 2). However, all these mitochondrial changes were clearly prevented in the HCT-116 BAX/BAK DKO cells, demonstrating a requirement for BAX and/or BAK for the perturbation of the mitochondria. Since BH3-only members are generally required to activate BAX and BAK, we wished to assess whether BH3 mimetics could induce mitochondrial structural perturbations in the absence of all known pro-apoptotic BH3-only members. For this, we used HCT-116 OctaKO cells, which lack the BH3-only members namely, BIM, BID, PUMA, BAD, BIK, HRK, BMF and NOXA^33^. Exposure of these cells to a combination of A-1331852 and A-1210477 resulted in mitochondrial structural changes, characteristic of significant cristae remodelling (Fig. 2). However, the swelling of mitochondrial matrix and the accompanying loss of cristae observed in the HCT-116 WT cells following BH3 mimetics were not apparent in HCT-116 OctaKO cells (Fig. 2). This is consistent with earlier findings demonstrating that the pro-apoptotic BH3-only members are dispensable for BH3 mimetic-mediated apoptosis^34^. Taken together, our data demonstrated that the activation of BAX and/or BAK, either in a BH3-dependent or independent manner, is essential for the ultrastructural changes observed in the mitochondria, following exposure to BH3 mimetics.

### DRP-1 is not required for the mitochondrial structural changes that occur during the onset of apoptosis

**Fig. 3:**
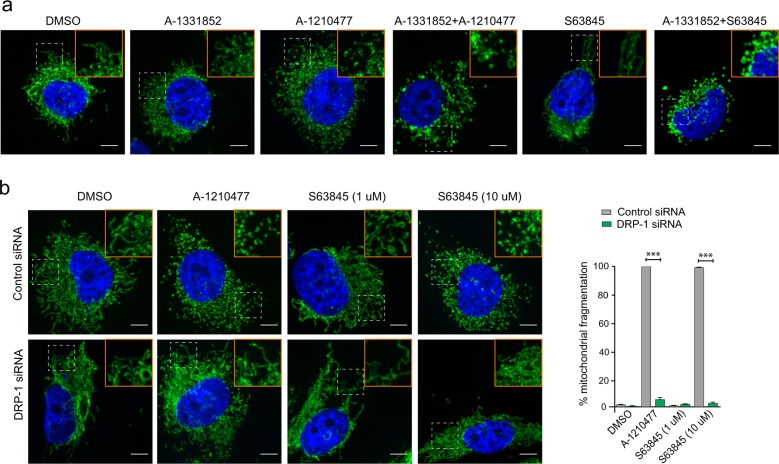
Mitochondrial fission mediated by A-1210477 and S63845 occurs in a DRP-1-dependent manner. **a** H1299 cells were exposed to Z-VAD.fmk (30 µM) for 0.5 h, followed by either A-1331852 (100 nM), A-1210477 (10 µM), S63845 (100 nM), or a combination of the different inhibitors for 4 h and assessed for mitochondrial integrity by immunostaining with HSP70 antibody. **b** H1299 cells were transfected with control or DRP-1 siRNA for 72 h and exposed to A-1210477 (10 µM) or S63845 (1 and 10 µM) for 4 h and assessed for mitochondrial integrity. The extent of mitochondrial fragmentation was quantified by analysing ~100 cells for each condition in three independent experiments. Scale bar: 10 µm. Error bars = mean ± SEM. Statistical analysis was conducted by one-way ANOVA (***P ≤ 0.001)

We previously reported that putative inhibitors of MCL-1 induced extensive mitochondrial fission and suggested that this could be a prerequisite for the ensuing apoptosis in MCL-1-dependent cell lines^25–27^. In support of this suggestion, exposure of A-1210477 but not A-1331852 resulted in extensive mitochondrial fission that resembled mitochondrial fragmentation in H1299 cells (Fig. 3a, Supplementary Fig. S2). The ability of A-1210477 to induce mitochondrial fission was also clearly evident when used in combination with A-1331852 to induce apoptosis in these cells (Fig. 3a, Supplementary Fig. S2). However, mitochondria in this instance appeared swollen, potentially indicating swollen matrix and loss of cristae that were previously observed at the level of electron microscopy (compare Figs. 1d and 3a). In marked contrast, S63845 at a concentration (100 nM) sufficient to induce apoptosis in a MCL-1-dependent manner^34^ failed to demonstrate mitochondrial fission (Fig. 3a, Supplementary Fig. S2). However, S63845 (100 nM) when used in conjunction with A-1331852 resulted in mitochondrial structural changes that resembled the swollen mitochondria observed following a combination of A-1210477 and A-1331852 (Fig. 3a, Supplementary Fig. S2). Taken together, our results suggested that mitochondrial fission mediated by A-1210477 versus a combination of MCL-1 and BCL-X_L_ inhibitors was distinct. Moreover, while S63845 failed to exhibit mitochondrial fission at low concentrations (100–1000 nM), higher concentrations (10 μM) of S63845 resulted in significant mitochondrial fission, which mimicked A-1210477-mediated mitochondrial fragmentation (Fig. 3b).

**Fig. 4:**
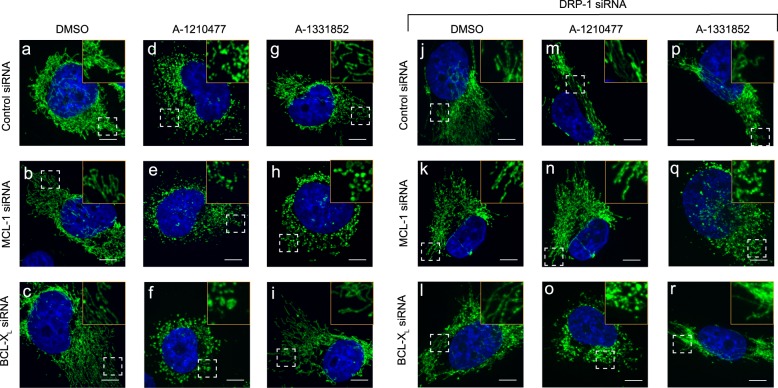
DRP-1 is not required for mitochondrial fission during BH3 mimetic-mediated apoptosis. H1299 cells were transfected with control, MCL-1, or BCL-X_L_ siRNAs, either alone or in combination with DRP-1 siRNA for 72 h, then exposed to Z-VAD.fmk (30 µM) for 0.5 h, followed by A-1210477 (10 µM) and/or A-1331852 (100 nM) for 4 h and assessed for mitochondrial integrity by immunostaining with HSP70 antibody. The boxed regions in the images are enlarged to show mitochondrial structural changes in the indicated cells. Scale bar: 10 µm

We previously reported that A-1210477-mediated mitochondrial fission occurred in a DRP-1-dependent manner^27^. A similar dependence on DRP-1 was also observed in cells exhibiting extensive mitochondrial fission, following exposure to high concentrations of S63845 (Fig. 3b). Thus both the MCL-1 inhibitors, A-1210477 and S63845, induced mitochondrial fission, which was clearly dependent on DRP-1 (Fig. 3b). We wished to assess if such mitochondrial fission was a prerequisite for apoptosis induction. Since H1299 cells depend on both BCL-X_L_ and MCL-1 for survival, we exposed cells to either A-1210477 or A-1331852 and simultaneously silenced the expression levels of either BCL-X_L_ or MCL-1 to facilitate apoptosis. Although downregulation of BCL-XL or MCL-1 did not result in mitochondrial fission and maintained the filamentous structure, exposure of the cells to A-1210477 resulted in significant mitochondrial fission, which resembled fragmented mitochondria (Fig. 4a–d, Supplementary Fig. S3). In the MCL-1-downregulated cells, A-1210477 still retained its ability to cause mitochondrial fragmentation (Fig. 4e), but when BCL-X_L_ was downregulated, A-1210477 resulted in mitochondrial structural changes that resembled matrix swelling (Fig. 4f, Supplementary Fig. S3), as previously described (Fig. 1d). In contrast, exposure to A-1331852 only resulted in similar mitochondrial swelling when MCL-1 was also downregulated (Fig. 4g–I, Supplementary Fig. S3). These results suggested that mitochondrial fission, mediated by MCL-1 inhibitors, appeared to exhibit a distinct morphology from that observed following the induction of apoptosis. This was more apparent following DRP-1 downregulation, which prevented A-1210477-mediated mitochondrial fission (Fig. 4m, n), but did not appear to alter mitochondrial swelling observed during apoptosis induction (Fig. 4o, q, Supplementary Fig. S3). Taken together, these results exclude an involvement of DRP-1 in the early mitochondrial structural changes including mitochondrial swelling associated with the onset of apoptosis (Fig. 4).

**Fig. 5:**
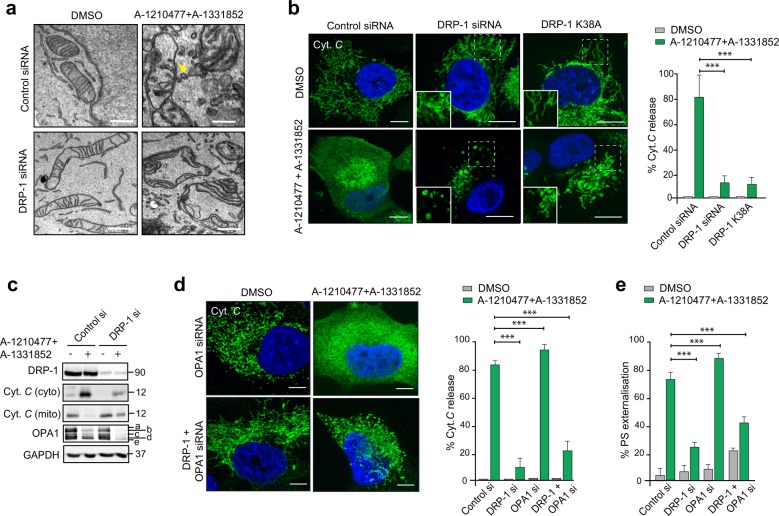
DRP-1 regulates BH3 mimetic-induced cytochrome *c* release and apoptosis downstream of mitochondrial cristae remodelling. **a** Electron microscopy of H1299 cells, transfected with DRP-1 siRNA for 72 h in the presence or absence of the indicated BH3 mimetics for 2 h. Breaks in the outer mitochondrial membrane are indicated by the yellow arrowhead. Scale bars = 10 nm. **b** H1299 cells were transfected with DRP-1 siRNA or GFP-DRP-1 K38A plasmid for 72 h, exposed to Z-VAD.fmk (30 µM) for 0.5 h, followed by a combination of A-1331852 (100 nM) and A-1210477 (10 µM) for 4 h and the extent of cytochrome *c* released from mitochondria assessed by confocal microscopy. The boxed regions in the images are enlarged to show mitochondrial structural changes in the indicated cells. The extent of cytochrome *c* release was quantified by counting at least 100 cells from three independent experiments. **c** Same as **b**, but the extent of cytochrome *c* release as well as OPA1 processing and the silencing efficiency of DRP-1 siRNA were analysed by western blotting. **d** H1299 cells were transfected with the indicated siRNAs for 72 h, treated as described in **b** and the extent of cytochrome *c* release assessed and quantified. The extent of cytochrome *c* release was quantified by counting at least 100 cells from three independent experiments. **e** Same as **d** but the cells were exposed to BH3 mimetics in the absence of Z-VAD.fmk and the extent of apoptosis assessed by PS externalisation from at least three independent experiments. All scale bars, unless indicated: 10 µm. Error bars = mean ± SEM. Statistical analysis was conducted by one-way ANOVA (***P ≤ 0.001)

Consistent with the above hypothesis, electron micrographs revealed marked structural alterations of the mitochondria in cells exposed to both A-121077 and A-1331852, characterised by breaks in the OMM (denoted by the yellow arrowheads), mitochondrial matrix swelling and a concomitant loss of cristae (Fig. 5a). Downregulation of DRP-1 alone resulted in elongated mitochondria, consistent with its known role in mitochondrial fission (Fig. 5a). However, the mitochondria in the DRP-1-downregulated cells following exposure to BH3 mimetics appeared visibly swollen with intact cristae and few if any breaks in the OMM (Fig. 5a). Taken together, our data suggested that mitochondrial fission observed following exposure to MCL-1 inhibitors was distinct from the structural perturbations (characterised by OMM breaks and IMM swelling) observed as a result of apoptosis induction.

### DRP-1 is critical for the release of cytochrome *c* from mitochondria during apoptosis

Permeabilisation of the OMM, otherwise known as MOMP, occurs as a consequence of BAX and/or BAK oligomerization and is generally accompanied by the release of mitochondrial cytochrome *c* into the cytosol. Exposure of cells to a combination of A-1210477 and A-1331852 resulted in an almost complete release of mitochondrial cytochrome *c* into the cytosol (Fig. 5b, c). This was markedly inhibited in cells, following inactivation of DRP-1 using siRNA or overexpression of the DRP-1 K38A plasmid (Fig. 5b, c), thus placing DRP-1 upstream of cytochrome *c* release. While cytochrome *c* was still retained in mitochondria following DRP-1 downregulation, mitochondria in these cells appeared swollen (Fig. 5b), consistent with those observed in the electron micrographs (Fig. 5a). As cytochrome *c* release occurs as a consequence of mitochondrial cristae remodelling^35^, exposure to BH3 mimetics not only resulted in the release of mitochondrial cytochrome *c* but also caused a loss of the high molecular weight isoforms of OPA1, characteristic of mitochondrial cristae remodelling (Fig. 5c). While downregulation of DRP-1 markedly diminished BH3 mimetic-mediated release of cytochrome *c*, it did not prevent BH3 mimetic-mediated loss of OPA1 (Fig. 5c), thus placing DRP-1 upstream of cytochrome *c* release but downstream of mitochondrial cristae remodelling. This was further confirmed following exposure of DRP-1 and/ or OPA1-downregulated cells to BH3 mimetics. While downregulation of OPA1 resulted in significant mitochondrial fission, as well as a near-complete release of cytochrome *c* following BH3 mimetics, a simultaneous downregulation of DRP-1 diminished these effects (Fig. 5d). Similarly downregulation of DRP-1 prevented BH3 mimetic-induced apoptosis, even in the absence of OPA1 (Fig. 5e), thus placing DRP-1 downstream of OPA1 proteolysis but upstream of cytochrome *c* release in BH3 mimetic-mediated apoptosis.

### DRP-1 is critical for BAK activation during BH3 mimetic-mediated apoptosis

**Fig. 6:**
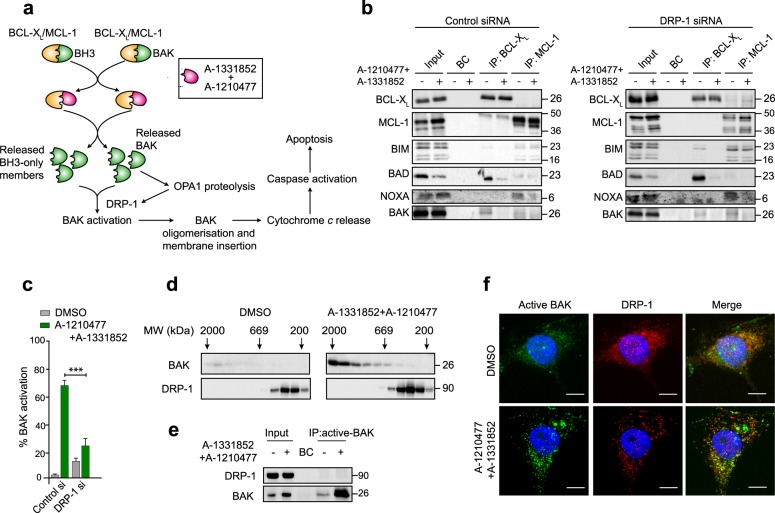
DRP-1 regulates BH3 mimetic-induced activation of BAK. **a** Scheme demonstrating the primary mechanism of action of BH3 mimetics and the downstream events that culminate in apoptosis. **b** Immunoprecipitation of BCL-X_L_ and MCL-1 was carried out in H1299 cells, transfected with control or DRP-1 siRNA, followed by exposure to Z-VAD.fmk (30 µM) for 0.5 h and a combination of A-1331852 (100 nM) and A-1210477 (10 µM) for 4 h, and the eluted complexes were immunoblotted for the indicated proteins. **c** H1299 cells were treated as **b** and the extent of BAK activation was assessed by flow cytometry from at least three independent experiments. Error bars = mean ± SEM. Statistical analysis was conducted by one-way ANOVA (***P ≤ 0.001). **d** Western blots of different molecular weight fractions from size exclusion chromatography showing BAK oligomerisation in H1299 cells upon exposure to A-1331852 (100 nM) and A-1210477 (10 µM) for 2 h. **e** Immunoprecipitation of active BAK in H1299 cells treated as **b**, and the eluted complexes were immunoblotted for the indicated proteins. **f** Representative images of cells showing BAK activation and DRP-1 distribution in H1299 cells treated as **b**. Scale bar: 10 µm

Our results indicated that BH3 mimetics could induce structural perturbations in the mitochondria, characterised by OPA1 proteolysis, cristae remodelling and the accompanying redistribution of cytochrome *c* from cristae to mitochondrial inner membrane space, all irrespective of the presence or absence of DRP-1. Since the role of DRP-1 was placed upstream of cytochrome *c* release, we wished to assess whether DRP-1 impacted on any upstream events during BH3 mimetic-mediated apoptosis. The primary function of BH3 mimetics is to disrupt the protein–protein interactions between the anti-apoptotic (BCL-X_L_ and MCL-1, in this instance) and pro-apoptotic members of the BCL-2 family. The released pro-apoptotic proteins could then activate the effector proteins (BAK, in H1299 as these cells lack BAX) to oligomerise on mitochondrial membranes to subsequently release cytochrome *c* (Fig. 6a). Immunoprecipitation of BCL-X_L_ and MCL-1 to identify their associated pro-apoptotic proteins revealed that in H1299 cells, BIM and BAK were bound to both BCL-X_L_ and MCL-1, whereas NOXA and BAD exclusively bound to MCL-1 and BCL-X_L_, respectively (Fig. 6b). Exposure of these cells to a combination of A-1331852 and A-1210477 resulted in displacement of most of these pro-apoptotic proteins from their corresponding ant-apoptotic partners (Fig. 6b). Importantly, none of these interactions/displacements were altered in cells following DRP-1 downregulation, thus suggesting that DRP-1 played no role in the early events of BH3 mimetic-mediated apoptosis. Since BAK and other BH3-only proteins were released following BH3 mimetics, we next wished to assess if BAK activation was altered in the absence of DRP-1. Downregulation of DRP-1 resulted in a significant decrease in BH3 mimetic-mediated activation of BAK (Fig. 6c), suggesting that DRP-1 was critical in the activation of BAK during BH3 mimetic-mediated apoptosis. Although the requirement of DRP-1 for BAK activation could be demonstrated, no binding of DRP-1 to the oligomerised/active BAK was observed in these cells (Fig. 6d–f), thus suggesting the involvement of other protein(s) in BAK activation immediately preceding cytochrome *c* release. Taken together, our data confirm that DRP-1 plays a critical role at the level of BAK activation, facilitating OMM breaks, cytochrome *c* release and apoptosis.

## Discussion

BH3 mimetics, in particular ABT-737 and ABT-199, induce a novel paradigm of cell death, characterised by excessive swelling of mitochondrial matrix and discontinuities in the OMM in BCL-2-dependent chronic lymphocytic leukaemia cells^19,31^. BH3 mimetics targeting BCL-X_L_ and MCL-1 also induce similar mitochondrial ultrastructural changes in cells that exclusively depend on BCL-X_L_ and MCL-1, respectively (Fig. 1)^20^. However, cells exposed to the MCL-1 inhibitor, A-1210477, exhibit marked mitochondrial changes, in particular mitochondrial fission, irrespective of their dependencies on a specific BCL-2 family member for survival (Fig. 3). This is in agreement with our previous findings^27^. Mitochondrial fission mediated by A-1210477 alone did not result in apoptosis in these cell lines, even after prolonged exposure^27^. This was most probably because most cell lines derived from solid tumours depend on both BCL-X_L_ and MCL-1 for survival, and inhibition of MCL-1 alone was not sufficient to result in apoptosis. This was further supported by our observation that inhibition of MCL-1 using A-1210477 while resulting in extensive mitochondrial fission did not induce OMM breaks and cell death, unless the activity of BCL-X_L_ was also neutralised.

Although A-1210477-mediated mitochondrial fission did not necessarily result in apoptosis, it was difficult to ascertain whether such fission was a prerequisite for apoptosis. This difficulty was partly because DRP-1 appeared to play important but distinct roles both in A-1210477-mediated mitochondrial fission and BH3 mimetic-mediated apoptosis^27^. Moreover, DRP-1 also interacted with MCL-1 and BCL-X_L_, thus coupling mitochondrial fission and apoptosis^23,27,36^. However, with the development of more potent inhibitors, such as S63845, we have demonstrated that mitochondrial fission does not occur at concentrations sufficient to inhibit MCL-1 (Fig. 3). Furthermore, while mitochondrial fission induced by A-1210477 and high concentrations of S63845 was mediated by DRP-1, mitochondrial swelling that occurred at the onset of apoptosis induction was largely independent of DRP-1 (Figs. 3 and 4), thus differentiating the distinct types of mitochondrial fission.

Our data in the HCT-116 WT and BAX/BAK DKO cells convincingly demonstrate that BH3 mimetic-mediated OMM breaks and swelling of matrix compartment are essential for BAX/BAK to facilitate cytochrome *c* release (Fig. 2). The inability of HCT-116 OctaKO cells to prevent BH3 mimetic-mediated mitochondrial changes further supports our findings that BAX and BAK but not the known BH3-only members are critical for BH3 mimetic-mediated apoptosis^33,34^. How BAX and BAK localise to the sites of OMM breaks to facilitate cytochrome *c* release is not entirely known. The involvement of DRP-1, Dynamin-2, and even membranes of the endoplasmic reticulum in these events have been previously proposed^37–42^. Downregulation of DRP-1 or its receptors, MID49 and MID51, have been shown to antagonise cytochrome *c* release and apoptosis in response to a wide variety of apoptotic stimuli^43^. DRP-1 functions downstream of OPA1-mediated cristae remodelling (Fig. 5), to activate BAK (Fig. 6), which in turn precedes BAK oligomerisation and membrane insertion for the execution of MOMP and apoptosis. However, mitochondrial cristae remodelling requires the presence of BAX and BAK (Fig. 2)^44^. Thus DRP-1 could function either downstream or independent of OPA1 proteolysis to activate BAK and ensuing apoptosis. Taken together, our data suggest that BH3 mimetics most likely activate BAX/BAK independently of the eight known BH3-only members, which further results in OPA1-mediated cristae remodelling to redistribute cytochrome *c* within the mitochondria, thus priming the mitochondria to undergo MOMP, upon sensing the stress signal. DRP-1 plays a critical role at this stage to activate BAK and/or BAX to insert these effector proteins on mitochondrial membranes. This along with the constriction of the primed mitochondria by DRP-1 constitute the so-called stress signals that cause OMM breaks, efficiently releasing the redistributed cytochrome *c* into the cytosol and initiating apoptosis.

## Materials and methods

### Cell culture

H1299 (purchased from ATCC), K562 (provided by Prof. R. Clark, University of Liverpool) and MAVER-1 cells (provided by Dr. J. Slupsky, University of Liverpool) were cultured in RPMI 1640 medium (Life Technologies). H929 cells (purchased from DMSZ, Braunshweig, Germany) were cultured in RPMI 1640 medium supplemented with 0.05 mM β-mercaptoethanol (BME). Colon cancer cell lines HCT-116 (wild-type and DKO) (from R. J. Youle, National Institute of Health, USA) and HCT-116-OctaKO^33^ were cultured in McCoy’s 5A Modified media. All culture media were supplemented with 10% FBS (Life Technologies) and maintained at 37 °C in a humidified atmosphere of 5% CO_2_. All cell lines used in this study were subjected to short tandem repeat (STR) profiling to ensure quality and integrity.

### Reagents

ABT-199, A-1210477 and Z-VAD.FMK from Selleck (Houston, TX, USA), S63845 from Active Biochem (Kowloon, Hong Kong) and A-1331852 from AbbVie Inc. (North Chicago, IL, USA) were used. Antibodies against HSP70 (cat#ab2799) from Abcam (Cambridge, UK), OPA1 (cat#612607), cytochrome *c* (cat#556432) and DRP-1 (cat#611113) from BD Biosciences (San Jose, CA, USA); BCL-X_L_ (cat#2762), BIM (cat#2933) and BAD (cat#9292) from Cell Signalling Technology (MA, USA); BAK (AB-1) (cat#AM-03) and NOXA (cat#OP180) from Millipore (Watford, UK) and MCL-1 (cat#sc-819), BAK (cat#sc-832) and GAPDH (cat#sc-25778) from Santa Cruz Biotechnologies (Santa Cruz, CA, USA) were used. All other reagents were obtained from Sigma Aldrich (St. Louis, MO, USA).

### Overexpression and genetic silencing

For transient overexpression studies, cells were transfected with GFP-DRP1 K38A plasmid (provided by Dr. E. Bampton, University of Leicester, UK), using TransIT-LT-1 transfection reagent (Mirus Bio LLC, Madison, WI, USA), according to the manufacturer’s protocol. For RNA interference, cells were transfected with 10 nM of siRNAs against DRP-1 (s104274235), MCL-1 (s8585 or SI02781205) or BCL-X_L_ siRNA (s1920) purchased from Qiagen Ltd (Manchester, UK) or ThermoFisher Scientific (Waltham, MA, USA). Cells were transfected using 0.33% (v/v) Interferin reagent (Polyplus Transfection Inc., NY) to culture media, according to the manufacturer’s protocol and processed 72 h after transfection.

### Microscopy

For electron microscopy, cells were fixed in 2.5% (w/v) glutaraldehyde and 2 mM calcium chloride in 0.1 M cacodylate buffer (pH 7.4). This was followed by heavy metal staining, which consisted of two consecutive osmium tetroxide steps (2% (w/v) OsO4 in ddh_2_O), followed by 1% (w/v) aqueous uranyl acetate. To prevent precipitation artefacts, the cells were washed copiously with ddH_2_O between each staining step. All fixation and staining steps were performed in a Pelco Biowave®Pro (Ted Pella Inc., Redding, California, USA) at 100w 20Hg, for 3 min and 1 min, respectively. Dehydration was in a graded ethanol series before filtration and embedding in medium premix resin (TAAB, Reading, UK). Seventy to 74 nm serial sections were cut using a UC6 ultra microtome (Leica Microsystems, Wetzlar, Germany) and collected on Formvar (0.25% (w/v) in chloroform (TAAB, Reading, UK) coated Gilder 200 mesh copper grids (GG017/C; TAAB, Reading, UK). Images were acquired on a 120 kV Tecnai G2 Spirit BioTWIN (FEI, Hillsboro, Oregon, USA) using a MegaView III camera and analySIS software (Olympus, Germany). For immunocytochemistry, cells grown on coverslips were fixed with 4% (w/v) paraformaldehyde, permeabilised with 0.5% (v/v) Triton X-100 in PBS, followed by incubations with primary antibodies (diluted 1:250 in 3% BSA in PBS), the appropriate fluorophore-conjugated secondary antibodies (diluted 1:1000 in 3% BSA in PBS), mounted on glass slides and imaged using a 3i Marianas spinning disk confocal microscope, fitted with a Plan-Apochromat ×63/1.4 NA oil objective, M27 and a Hamamatsu ORCA-Flash4.0 v2 sCMOS Camera (all from Intelligent Imaging Innovations, GmbH, Germany).

### Cytochrome *c* release assay

Approximately 10^6^ cells were washed in cold PBS and resuspended in mitochondrial isolation buffer (250 mM sucrose, 20 mM HEPES, pH 7.4, 5 mM MgCl_2_ and 10 mM KCl) containing 0.01% digitonin. Cells were left on ice for 5 min followed by centrifugation at 13000 *g* for 3 min at 4 °C. Subsequently, the supernatant (cytosolic fraction) and pellet (mitochondrial fraction) were processed for western blotting.

### Size exclusion chromatography, immunoprecipitation and western blotting

Size exclusion chromatography and immunoprecipitation experiments were carried out as previously described^16,27^. Western blotting was carried out according to standard protocols. Briefly, 50 μg of total protein lysate was subjected to SDS-PAGE electrophoresis. Subsequently proteins were transferred to nitrocellulose membrane, probed with appropriate primary antibodies (diluted 1:1000 in Tris-buffered saline with 0.1% Tween-20), species-specific secondary antibodies (diluted 1:2000 in Tris-buffered saline with 0.1% Tween-20) and protein bands visualised with ECL reagents (GE Healthcare).

### Flow cytometry

The extent of apoptosis in cells following different treatments was quantified by using an Attune NxT flow cytometer (ThermoFisher Scientific, Paisley, UK) following staining of the cells with AnnexinV-FITC and propidium iodide to measure phosphatidylserine externalisation, as previously described^45^. Loss in mitochondrial membrane potential (*ψ*_m_) was assessed as described previously^19^ by staining cells with TMRE, a lipophilic fluorescent dye that accumulates in the mitochondria in relation to the membrane potential, and quantified by flow cytometry. For BAK activation, cells were fixed with 2% paraformaldehyde at room temperature for 10 min, washed with PBS and resuspended in a buffer containing 0.1% saponin and 0.5% BSA in PBS for 10 min. The cell suspension was then incubated with 0.1 mg/ml of anti-BAK AB-1 (Calbiochem Research Biochemicals—now Merck, cat#AM-03) antibody for 1 h at 4 °C, followed by further incubation with goat-anti-mouse IgG-AlexaFluor-488 conjugated secondary antibody for 1 h at 4 °C, before being subjected to flow cytometry.

### Statistical analysis

Statistical analysis was conducted by using one-way ANOVA with Bonferroni’s multiple comparison test was performed to evaluate differences between numerical variables. Asterisks depicted correspond to the following *p* values: *p *≤* 0.05, **p ≤ 0.005 and ***p *≤* 0.001.

## Supplementary information

Figure S3

Supplemental legends

Figure S1

Figure S2
