# Supplementary figures and images for: DRP-1 functions independently of mitochondrial structural perturbations to facilitate BH3 mimetic-mediated apoptosis
Source: Cell Death Discov. 2019 Jul 17;5:117. doi: 10.1038/s41420-019-0199-x (PMC6637195; doi:10.1038/s41420-019-0199-x)

# Figure S3

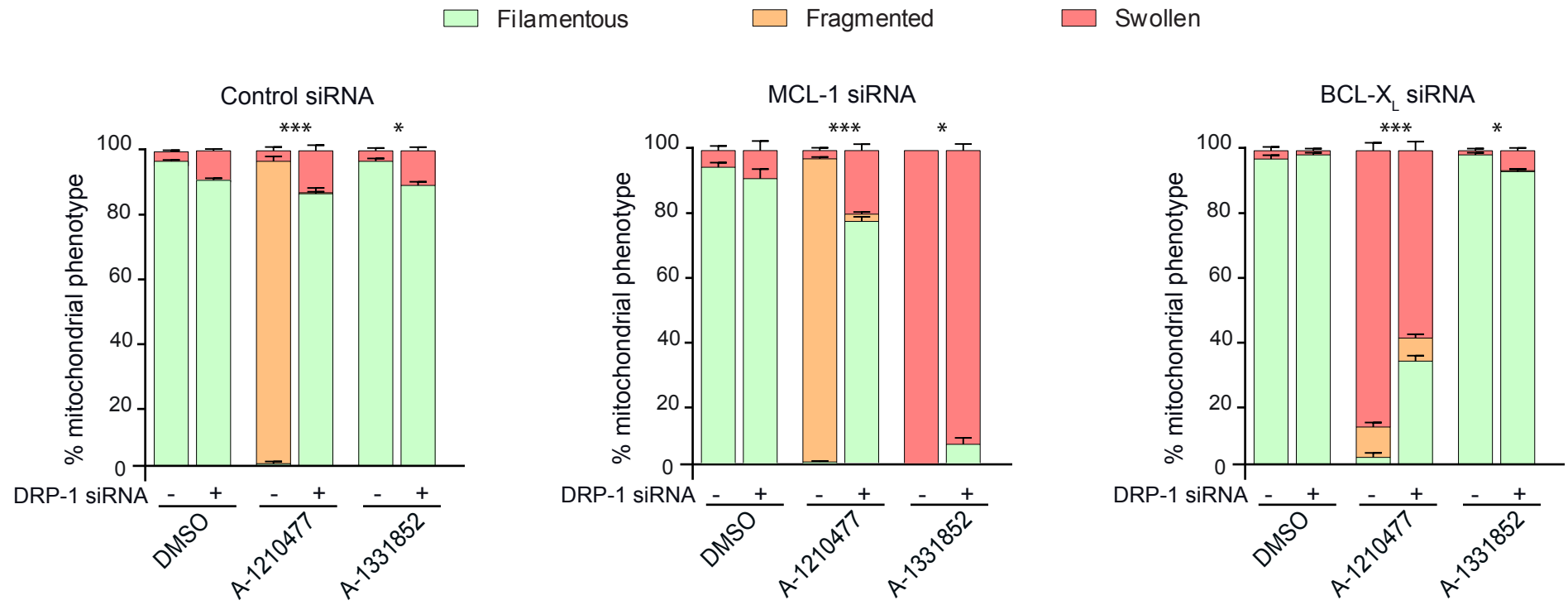

Supplement: Supplementary file 1 — Figure S3 [file 41420_2019_199_MOESM1_ESM.pdf]

# Figure S1

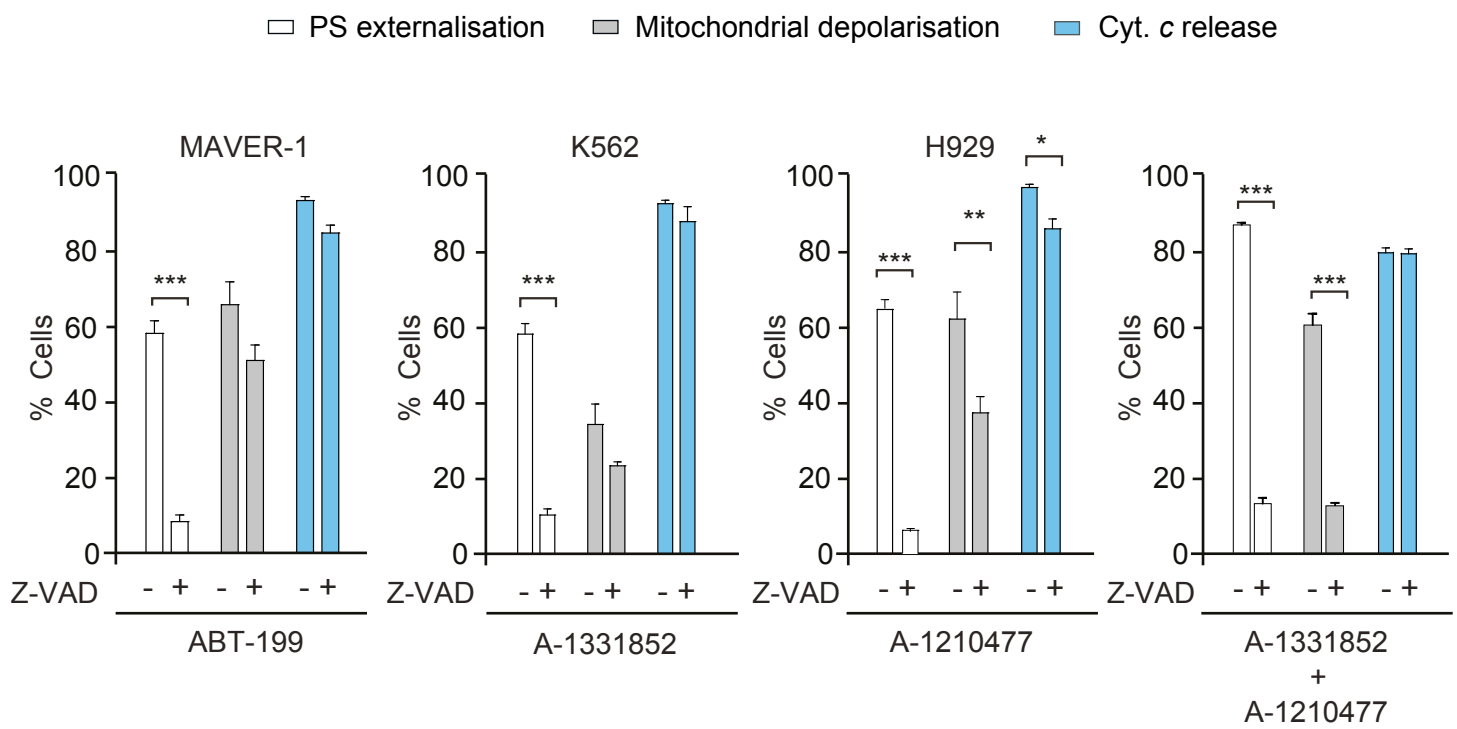

Supplement: Supplementary file 3 — Figure S1 [file 41420_2019_199_MOESM3_ESM.pdf]

Figure S2

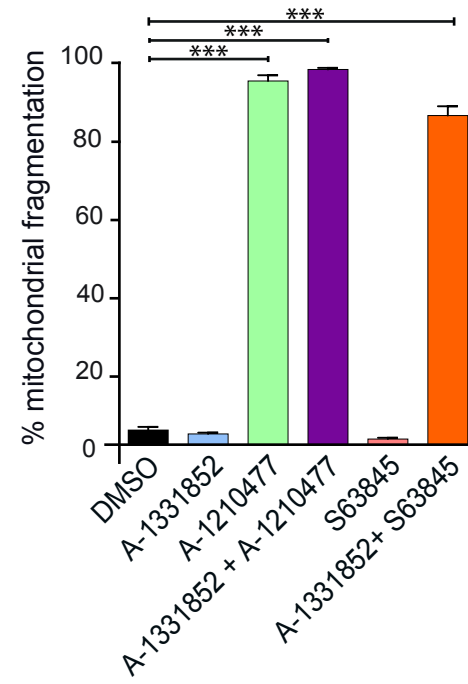

Supplement: Supplementary file 4 — Figure S2 [file 41420_2019_199_MOESM4_ESM.pdf]
