# Supplementary material for: DRP-1 functions independently of mitochondrial structural perturbations to facilitate BH3 mimetic-mediated apoptosis
Source: Cell Death Discov. 2019 Jul 17;5:117. doi: 10.1038/s41420-019-0199-x (PMC6637195; doi:10.1038/s41420-019-0199-x)
Supplement: Supplementary file 2 — Supplemental legends [file 41420_2019_199_MOESM2_ESM.docx]

**Supplementary Figure Legends**

**Fig. S1. Externalisation of phosphatidylserine, but not mitochondrial depolarisation or MOMP, occurs in a caspase-dependent manner.**  MAVER-1, K562, H929 and H1299 cells were exposed to Z-VAD.fmk (30 µM) for 0.5 h, followed by ABT-199 (100 nM), A-1331852 (100 nM), A-1210477 (10 µM) or a combination of A-1331852 (100 nM) and A-1210477 (10 µM), respectively for 4 h and assessed for the extent of phosphatidylserine (PS) externalisation, loss in mitochondrial membrane potential and cytochrome *c* release. Graphs for cytochrome *c* release were plotted by counting at least 100 cells from three independent experiments. Error bars = Mean ± SEM. Statistical analysis was conducted by one-way ANOVA (* *p* ≤ 0.05, ** *p* ≤ 0.005 and *** *p* ≤ 0.001).

**Fig. S2. Mitochondrial fragmentation is induced by BH3 mimetic-mediated inhibition of MCL-1 and BCL-X_L_.** H1299 cells were exposed to Z-VAD.fmk (30 µM) for 0.5 h, followed by A-1331852 (100 nM), A-1210477 (10 µM), a combination of A-1331852 (100 nM) and A-1210477 (10 µM), S63845 (100 nM), or a combination of S63845 (100 nM) and A-1331852 (100 nM), for 4 h and assessed for mitochondrial integrity by immunostaining with HSP70 antibody. The extent of mitochondrial fragmentation was quantified by analysing ~100 cells for each condition in three independent experiments. Error bars = Mean ± SEM. Statistical analysis was conducted by one-way ANOVA (****P*≤0.001).

**Fig S3. BH3 mimetic-mediated mitochondrial fragmentation but not mitochondrial swelling is regulated by DRP-1.** H1299 cells were transfected with control, MCL-1 or BCL-X_L_ siRNAs, either alone or in combination with DRP-1 siRNA for 72 h, then exposed to Z-VAD.fmk (30 µM) for 0.5 h, followed by A-1210477 (10 µM) and/or A-1331852 (100 nM) for 4 h and assessed for mitochondrial integrity by immunostaining with HSP70 antibody. The extent of mitochondrial fragmentation was quantified by analysing ~100 cells for each condition in three independent experiments and the percentage of different mitochondrial phenotypes, filamentous, fragmented or swollen was calculated. Error bars = Mean ± SEM. Statistical analysis was conducted by one-way ANOVA (****P*≤0.001).
